# *Journal of Synchrotron Radiation* welcomes seven new Co-editors

**DOI:** 10.1107/S1600577525009361

**Published:** 2025-10-30

**Authors:** Dibyendu Bhattacharyya, Kristina Kvashnina, Makina Yabashi

**Affiliations:** a Atomic and Molecular Physics Division Bhabha Atomic Research Centre Mumbai400085 India; b Rossendorf Beamline, ESRF – The European Synchrotron, 71 Avenue des Martyrs, 38000Grenoble, France; c Beam Line Research and Development Group, XFEL Research and Development Division, RIKEN SPring-8 Center, 1-1-1 Kouto, Mikazuki-cho, Sayo-gun, Hyogo679-5148, Japan

**Keywords:** Co-editors, synchrotron radiation, free-electron lasers

## Abstract

The newest seven members of the Editorial Board of *Journal of Synchrotron Radiation* are introduced.

*Journal of Synchrotron Radiation* continues to welcome articles covering the entire field of synchrotron radiation and X-ray free-electron laser research. We are excited to strengthen our Editorial Board with the addition of seven new Co-editors. This expansion will ensure we continue to cover the full spectrum of topics relevant to our readers, while also addressing important new and emerging areas within the field. ▸

*Dr Sharon Bone* is a Staff Scientist at Forschungszentrum Jülich, Germany, who’s interest include X-ray absorption spectroscopy (EXAFS, XANES), X-ray spectromicroscopy (µ-XRF, STXM), biogeochemistry and environmental engineering.

*Professor Yan-Gu Lin* is a Fellow of the Royal Society of Chemistry from National Synchrotron Radiation Research Center, Taiwan, with expertise in X-ray absorption, emission and photoelectron spectroscopy, *in situ*/*operando* experiments, X-ray diffraction and scattering, and energy and catalysis science.

*Professor Anders Madsen* is Leading Scientist and Group Leader of Materials Imaging and Dynamics at the European X-ray Free-Electron Laser Facility in Schenefeld, Germany, with expertise in X-ray free-electron lasers, coherent scattering and imaging, soft matter and liquid surfaces, X-ray photon correlation spectroscopy and X-ray instrumentation.

*Dr Elke Plönjes* is Group Leader of the FLASH Beamlines and Optics group at DESY, Germany, with interest including X-ray free-electron lasers, X-ray instrumentation and beamline development and simulation, X-ray coherence and ptychography, and has worked on the journal before as Guest Editor on several special issues.

*Dr Marius Retegan* is a Scientific Software Developer at The European Synchrotron Radiation Facility, France, who’s areas of expertise include computational spectroscopy, software development, artificial intelligence, X-ray absorption and emission spectroscopy, and resonant inelastic X-ray scattering.

*Professor Changyong Song* is Professor of the Department of Physics at Pohang University of Science and Technology (POSTECH) in Pohang, South Korea, with interests including ultrafast time-resolved imaging and diffraction, coherent imaging and scattering, X-ray free-electron lasers and X-ray coherence.

*Professor Zhonghua Wu* is Senior Researcher at Institute of High Energy Physics (IHEP), Professor of University of Chinese Academy of Sciences (CAS), and Principal of the XRD and SAXS experimental stations of Beijing Synchrotron Radiation Facility (BSRF), China, and an expert in X-ray absorption spectroscopy, powder diffraction, small-angle X-ray scattering, beamline instrumentation, and *in situ* and *operando* techniques.

The addition of these new Co-editors to the journal’s Editorial Board brings fresh expertise to improve existing research areas and explore new ones. This also increases the representation of different institutions and geographical regions globally.

## Figures and Tables

**Figure 1:**
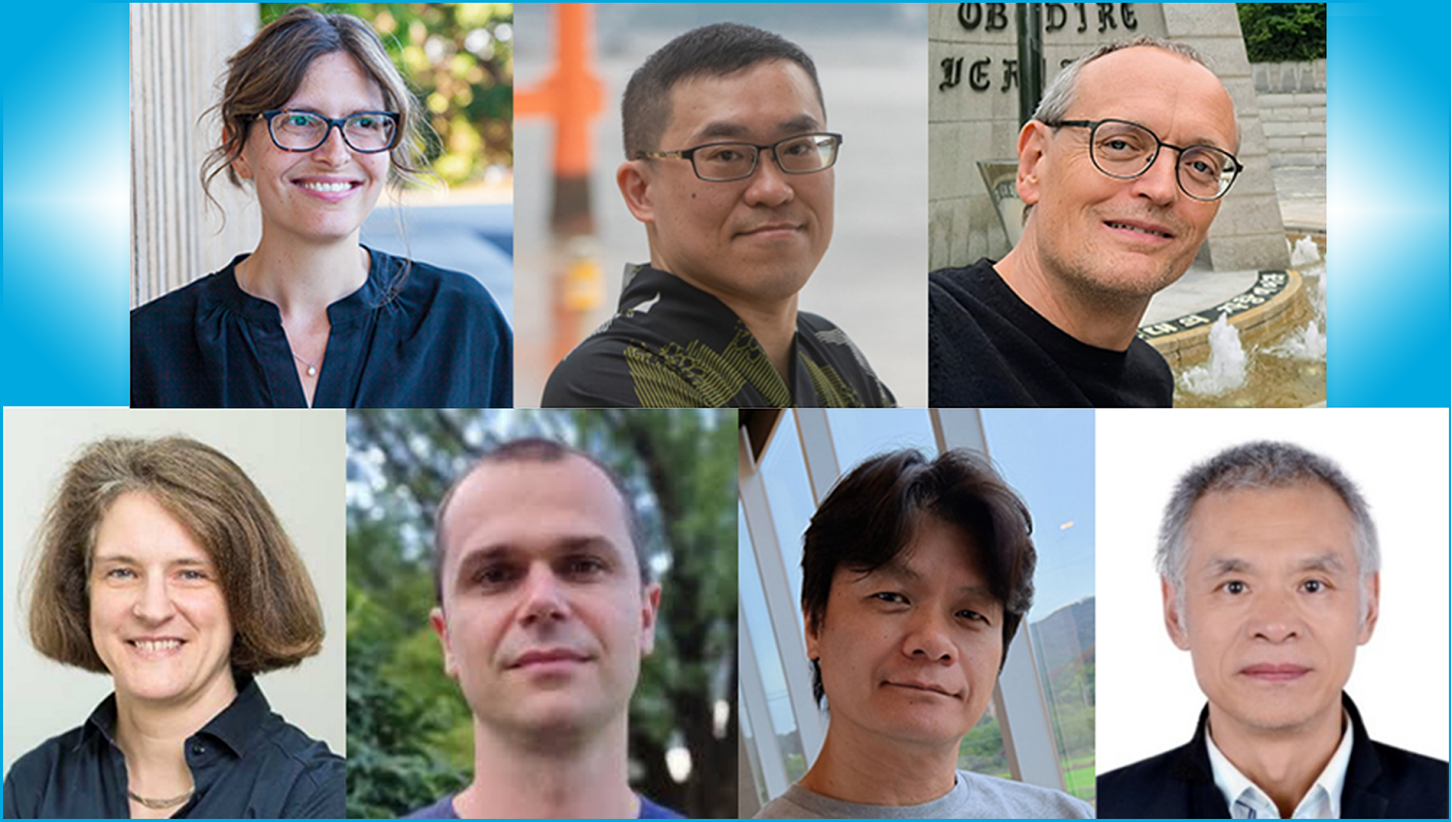
Our new Co-editors, (top, left to right) Sharon Bone, Yan-Gu Lin, Anders Madsen, (bottom, left to right) Elke Plönjes, Marius Retegan, Changyong Song and Zhonghua Wu.

